# Metformin improves endotoxemia and alters folliculogenesis in women with polycystic ovary syndrome

**DOI:** 10.1530/EC-26-0120

**Published:** 2026-05-26

**Authors:** Mateusz Trzcinski, Izabela Chudzicka-Strugala, Katherine Kim, Piotr Piekarski, Robert Spaczynski, Barbara Zwoździak, Beata Banaszewska, Leszek Pawelczyk, R Jeffrey Chang, Antoni J Duleba

**Affiliations:** ^1^ Department of Gynecological Endocrinology and Infertility Treatment, Department od Diagnostics and Treatment of Infertility, Poznan University of Medical Sciences, Poznan, Poland; ^2^ Department of Medical Microbiology, Poznan University of Medical Sciences, Poznan, Poland; ^3^ Division of Reproductive Endocrinology and Infertility, Department of Obstetrics, Gynecology and Reproductive Sciences, University of California San Diego, La Jolla, California, USA; ^4^ Department of Maternal and Child Health and Minimally Invasive Surgery, Poznan University of Medical Sciences, Poznan, Poland; ^5^ Collegium Medicum, University of Zielona Góra, Zielona Góra, Poland; ^6^ Department of Laboratory Diagnostics, Poznan University of Medical Sciences, Poznan, Poland

**Keywords:** polycystic ovary syndrome, metformin, ovarian morphology, endotoxemia, testosterone

## Abstract

**Abstract:**

Polycystic ovary syndrome (PCOS) is associated with excessive ovarian androgen production, an increased number of ovarian follicles and a wide range of other endocrine and metabolic derangements, including endotoxemia. Metformin is often used in the treatment of PCOS, especially to improve glucose metabolism. This study evaluated the effects of metformin on endotoxemia, folliculogenesis and endocrine profiles. In a prospective trial, women with PCOS received metformin (500 mg t.i.d.) for three months and underwent baseline and post-treatment evaluations of their endocrine and metabolic profiles as well as detailed ultrasonographic evaluations of ovaries. Metformin treatment was associated with reduced lipopolysaccharides (LPSs) by 19% (*P* < 0.0001) and LPS-binding protein (LPB) by 26% (*P* < 0.0001). In parallel, the number of small antral follicles (<6 mm) declined by 8% (*P* = 0.005), total testosterone decreased by 13% (*P* = 0.0003), ovarian testosterone production in response to hCG declined by 73% (*P* = 0.03), and fasting insulin decreased by 19% (*P* = 0.02). The reduction in testosterone following treatment with metformin may be due to a combination of multiple effects of metformin, including improvement in endotoxemia, reduction in folliculogenesis and decreased insulin.

**Clinical trial registration number:**

NCT03489668

## Introduction

Polycystic ovary syndrome (PCOS) is the most common endocrinopathy affecting women in reproductive age (1, 2, 3); however, its etiology and pathophysiology remain poorly understood. Key features of this syndrome are excessive ovarian androgen production, oligo- or anovulation and altered ovarian morphology characterized by altered folliculogenesis leading to increased number of small and medium follicles. Furthermore, a large proportion of women with PCOS suffer from a wide range of metabolic derangements, including insulin resistance leading to compensatory hyperinsulinemia, dyslipidemia and systemic inflammation. One potential explanation tying together most of the above derangements has been proposed by Tremellen and Pearce in 2012 as DOGMA theory, postulating that dysbiosis of gut microbiome may be a major contributor to the pathophysiology of PCOS (4). Indeed, consistent with this theory, we and other investigators found that PCOS is characterized by gut dysbiosis (5, 6, 7, 8). Several studies demonstrated that gut microbiome of women with PCOS has increased abundance of gram-negative bacteria (e.g. Bacteroides) (6, 7). Recently, we have found that PCOS is also associated with endotoxemia, a condition defined as an elevation in the level of lipopolysaccharides (LPSs) in systemic circulation (9). We also found that in cultures of ovarian theca–interstitial cells, LPSs induce significant concentration-dependent stimulation of growth and androgen production by these cells (10). These effects of LPSs were accompanied by a profound shift in gene expression; in particular, there was a significant increase in the expression of key genes involved in the biosynthesis of cholesterol and androgens, including *Cyp17a1*, *Cyp11a1*, *Hsd3b* and *Hmgcr.*

In view of the above considerations, it is likely that PCOS may be improved by reduction in LPSs either by modification of gut microbiome or by decreasing gut permeability. One potential agent that may have such effects is metformin. Metformin is commonly used to treat PCOS, and several studies, including meta-analyses, have demonstrated its beneficial effects, including reduction in androgen, improvement in insulin resistance and a decrease in the number of antral ovarian follicles (11, 12, 13, 14, 15, 16, 17). Furthermore, metformin was shown to counteract pro-inflammatory effects of LPSs, as demonstrated by various *in vitro* and *in vivo* studies (18, 19, 20, 21). Novel animal *in vivo* and *in vitro* studies demonstrated that metformin may also exert beneficial effects on gut epithelial barrier integrity and may protect against intestinal barrier dysfunction (22, 23); consequently, metformin may reduce influx of bacteria or bacterial fragments, such as cell walls of gram-negative bacteria containing LPSs. Clinical trials demonstrated that metformin may also alter gut microbiome (24, 25). These observations prompted the present study to evaluate the effects of metformin on endotoxemia as well as to assess its effects on other features of PCOS, including ovarian hyperandrogenism and polycystic ovarian morphology.

## Materials and methods

### Subjects

**Figure 1:**
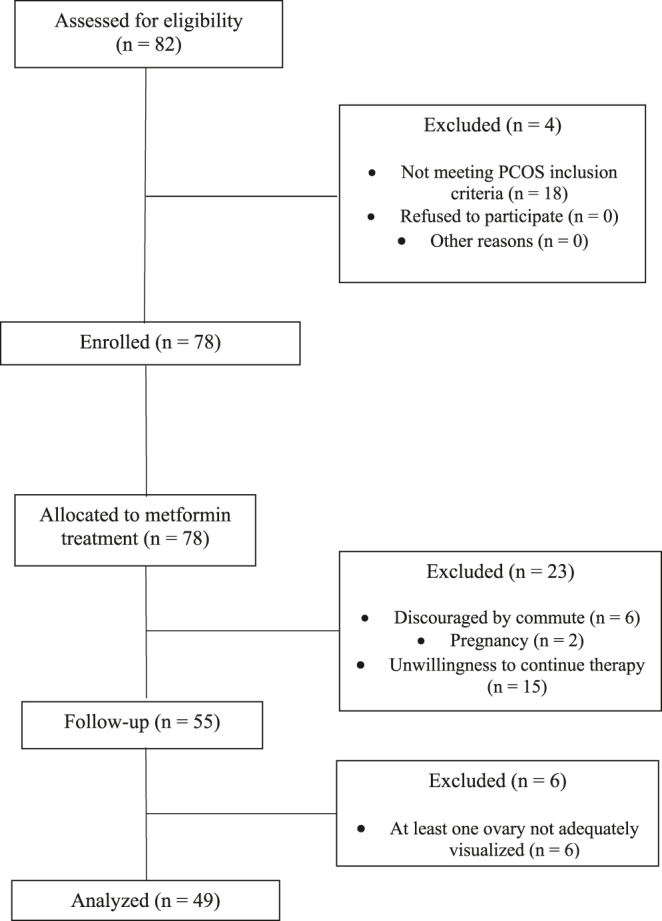
Diagram summarizing the design, enrollment, randomization, and follow-up of subjects in this study (CONSORT flow diagram).

The study was performed at a single academic center at Poznan University of Medical Sciences. The CONSORT flow chart summarizes the progress of participants through the study (Fig. 1). PCOS was defined according to the Rotterdam Criteria based on the presence of at least two of the following features: i) clinical or chemical hyperandrogenism (Ferriman–Gallwey score ≥ 8 and/or total testosterone > 0.5 ng/mL), ii) oligo- or anovulation (<8 spontaneous cycles per year) and/or iii) polycystic ovarian morphology as determined by transvaginal ultrasound (26). Other endocrine disorders, such as diabetes mellitus, thyroid disease, hyperprolactinemia and Cushing’s disease, were excluded. The study was approved by the institutional review board, and all participants signed written consent. As presented in Fig. 1, the study was completed by 71% of enrolled subjects; additional six subjects were excluded from final analysis due to inability to obtain complete evaluation of both ovaries at the time of follow-up.

### Procedures

Consenting participants were evaluated at the baseline during the follicular phase of the natural menstrual cycle or following medroxyprogesterone-induced menses; repeat evaluations were performed again after three months of treatment with metformin (500 mg t.i.d.).

### Clinical parameters

Clinical assessments consisted of determinations of weight and height (to calculate body mass index, BMI), measurements of waist and hip circumference (to calculate waist-to--hip ratio, WHR), Ferriman–Gallwey hirsutism score (27) and acne score (four-point scale described previously (11)). Detailed transvaginal evaluations of ovaries were carried out using GE Voluson E8 Expert (USA). Ovarian volumes were calculated using the prolate ellipsoid formula. Serum specimens were stored frozen at −70°C until analysis.

### Endocrine parameters

The capacity of theca cells to synthesize androgen was determined with the aid of the hCG challenge test whereby the testosterone level was determined before and 24 h after administration of hCG (25 mg, i.v.). Testosterone, LH, FSH, SHBG and DHEAS levels were determined by electroluminescence assays using COBAS e601 (Roche Diagnostic, Switzerland).

### Metabolic parameters

LPSs were quantified using the Limulus *Amebocyte Lysate* chromogenic assay (Hycult Biotech, The Netherlands). The minimum detection limit of this assay was 0.04 EU/mL, and a measurable concentration range was from 0.04 to 10 EU/mL. LPS-binding protein (LBP) was quantified using a human LBP enzyme-linked immunosorbent assay kit (LBP ELISA; Hycult Biotech, The Netherlands). This assay has a detection range from 4.4 ng/mL up to 50 ng/mL. Oral glucose tolerance test and insulin determinations were performed by testing glucose and insulin in the fasting state and subsequently at 30, 60, 90 and 120 min after glucose load (75 g, p.o.). Glucose levels were determined using the enzymatic reference method with hexokinase (Cobas c501, Roche Diagnostics Polska, ROCHE Holding AG, Switzerland). The insulin sensitivity index (ISI) was derived using glucose and insulin levels obtained during an oral glucose tolerance test as described by Matsuda and DeFronzo: Insulin sensitivity index = (10,000/square root of ((fasting glucose × fasting insulin) × (mean glucose × mean insulin during oral glucose tolerance test)) (28). Total cholesterol and triglycerides were quantified using enzymatic colorimetric assays (Cobas c501, Roche Diagnostics Polska, ROCHE Holding AG, Switzerland). High-density lipoprotein (HDL) was separated by precipitating apolipoprotein B (Roche Polska sp z o.o. Poland. Low-density lipoprotein cholesterol (LDL-C) was calculated using the Friedewald formula.

### Statistical analysis

The data were analyzed with the aid of JMP Pro 17 statistical software (SAS Institute, USA). *P*-values <0.05 were considered significant. Comparisons of baseline and follow-up values were performed using paired *t*-test, or in the absence of a normal distribution (evaluated by the Shapiro–Wilk test), following Box–Cox transformations or employing non-parametric testing (the Wilcoxon signed-rank test).

## Results

The study was completed by 71% of enrolled subjects, but additional six subjects were excluded from the final analysis due to inability to obtain complete evaluation of both ovaries at the time of follow-up.

Table 1 summarizes effects of a three-month course of metformin on clinical, endocrine and metabolic parameters relevant to PCOS. Clinically, metformin treatment was associated with a modest but statistically significant reduction in BMI by 3.6% and an improvement in hirsutism by 2.4%. In parallel, total T declined by 13% and theca cell responsiveness to hCG (change in T level over 24 h) decreased by 73%. These changes occurred in the absence of significant changes in DHEAS or gonadotropins. Metformin treatment was also associated with significant alterations in measures of endotoxemia as evidenced by a 21% decrease in serum levels of LPSs and a 26% reduction in the serum LBP. Other significant metabolic changes included a 19% reduction in fasting insulin and a modest 4% decrease in LDL cholesterol.

**Table 1:** Clinical, endocrine, and metabolic characteristics in women with PCOS (*n* = 49) before and after metformin treatment (1,500 mg/day for 3 months).

Variable	Baseline	At 3 months	Total change (3 − 0 months)	*P*-value (baseline vs 3 months)
**Clinical parameters**				
BMI (kg/m^2^)	28.4 ± 1.0	27.4 ± 0.94	−1.0 ± 0.2	*P* < 0.0001
WHR	0.81 ± 0.009	0.82 ± 0.008	0.006 ± 0.005	NS
Hirsutism (Ferriman–Gallwey score)	11.5 ± 0.89	11.2 ± 0.89	−0.27 ± 0.07	*P* = 0.0005
Acne (score)	0.92 ± 0.15	0.92 ± 0.14	0 ± 0.09	NS
**Endocrine parameters**				
AMH (ng/mL)	9.56 ± 0.92	8.95 ± 0.82	−0.61 ± 0.35	NS
T (0 h) (ng/mL)	0.62 ± 0.03	0.54 ± 0.02	−0.081 ± 0.02	*P* = 0.0003
T (24 h) (ng/mL)	0.68 ± 0.04	0.63 ± 0.03	−0.04 ± 0.02	NS
Δ T (24 − 0 h) (ng/mL)*	0.37 ± 0.42	0.09 ± 0.01	−0.27 ± 0.41	*P* = 0.03
DHEAS (μmol/L)	8.99 ± 0.48	9.19 ± 0.55	0.20 ± 0.32	NS
SHBG (nmol/L)	51.3 ± 5.82	49.6 ± 5.00	−1.65 ± 2.77	NS
LH (mIU/mL)	11.0 ± 0.86	10.4 ± 0.90	−0.58 ± 0.66	NS
FSH (mIU/mL)	5.16 ± 0.22	4.89 ± 0.27	−0.27 ± 0.28	NS
LH:FSH ratio	2.16 ± 0.16	1.17 ± 1.29	−0.99 ± 1.28	NS
Prolactin (μg/L)	20.1 ± 1.02	21.1 ± 1.20	1.27 ± 1.00	NS
**Metabolic parameters**				
LPSs (EU/mL)	5.23 ± 0.30	4.07 ± 0.20	−1.11 ± 0.23	*P* < 0.0001
LBP (μg/mL)	39.1 ± 1.60	28.8 ± 1.02	−9.99 ± 0.86	*P* < 0.0001
Insulin fasting (μU/mL)	16.7 ± 2.01	13.5 ± 1.37	−3.15 ± 1.25	*P* = 0.02
Glucose fasting (mg/dL)	91.4 ± 1.62	90.0 ± 1.15	−1.41 ± 1.48	NS
ISI	3.50 ± 0.35	3.29 ± 0.25	−0.21 ± 0.28	NS
Total cholesterol (mg/dL)	161.9 ± 3.95	157.4 ± 3.37	−4.52 ± 3.49	NS
LDL cholesterol (mg/dL)	90.7 ± 3.44	87.2 ± 3.17	−3.50 ± 2.70	*P* = 0.04
HDL cholesterol (mg/dL)	52.2 ± 2.14	52.9 ± 1.93	0.70 ± 1.23	NS
Triglycerides (mg/dL)	97.0 ± 7.05	96.9 ± 11.3	−0.10 ± 7.94	NS

Baseline and follow-up comparisons were performed using paired *t*-tests or, in the absence of normal distribution, by the Wilcoxon signed-rank test.

Data are expressed as mean ± standard error.

NS = not significant.

* Change in the parameter at 24 h after administration of hCG vs the baseline (prior administration of hCG).

17-OHP, 17-hydroxyprogesterone; AUC, area under the curve; BMI, body mass index; DHEAS, dehydroepiandrosterone sulfate; E2, estradiol; FSH, follicle-stimulating hormone; LBP, lipopolysaccharide-binding protein; LDL, low-density lipoprotein; LH, luteinizing hormone; LPSs, lipopolysaccharides; HDL, high-density lipoprotein; ISI, insulin sensitivity index; P4, progesterone; PCOS, polycystic ovary syndrome; SHBG, sex hormone-binding globulin; T, testosterone; WHR, waist-to-hip ratio.

A detailed evaluation of ovarian morphology by ultrasound at baseline and at the conclusion of the study is described in Table 2. The total number of measurable antral follicles declined by 7% due to a decrease in the number of follicles measuring less than 6 mm by 8%. Statistically non-significant decreases in the number of follicles were noted in each subpopulation of small follicles measuring <2 mm, 2–3.9 mm, and 4–5.9 mm.

**Table 2:** Ovarian morphology in women with PCOS (*n* = 49) before and after metformin.

Variable	Baseline	At 3 months	Total change (3 − 0 m)	*P*-value
Total ovarian volume (mL)	26.2 ± 1.5	24.0 ± 1.1	−2.2 ± 0.9	NS
Total number of follicles	153.4 ± 9.5	143.2 ± 9.8	−10.2 ± 6.5	*P* = 0.004
Total number of follicles grouped by size				
<2 mm	33.9 ± 4.82	29.7 ± 5.10	−4.2 ± 3.5	NS
2–3.9 mm	86.6 ± 6.4	81.5 ± 6.9	−5.1 ± 5.8	NS
4–5.9 mm	28.2 ± 2.4	26.3 ± 1.8	−1.9 ± 0.6	NS
6–7.9 mm	4.1 ± 0.5	4.6 ± 0.5	0.5 ± 0.6	NS
8–9.9 mm	0.5 ± 0.12	0.8 ± 0.17	0.3 ± 0.2	NS
10–11.9 mm	0.1 ± 0.04	0.20 ± 0.1	0.1 ± 0.1	NS
12–13.9 mm	0.00 ± 0.00	0.06 ± 0.03	0.06 ± 0.03	NS
<4 mm	120.5 ± 8.40	111.1 ± 9.2	−9.4 ± 6.9	*P* = 0.02
<6 mm	148.7 ± 9.6	137.4 ± 9.8	−11.3 ± 6.6	*P* = 0.005
≥6 mm	4.8 ± 0.5	5.8 ± 0.6	1.00 ± 0.7	NS
≥10 mm	0.2 ± 0.1	0.4 ± 0.1	0.2 ± 0.1	NS
≥14 mm	0.08 ± 0.04	0.16 ± 0.05	0.08 ± 0.06	NS

Baseline and follow-up comparisons were performed using paired *t*-tests or, in the absence of normal distribution, by the Wilcoxon signed-rank test.

Data are expressed as mean ± standard error.

NS = not significant.

## Discussion

This study demonstrated that administration of metformin to women with PCOS led to significant improvements in several key features of PCOS, including ovarian hyperandrogenism, parameters of endotoxemia, fasting insulin and the number of antral follicles measuring less than 6 mm. The metformin-induced improvement in hyperandrogenism is characterized by a reduction in the level of testosterone and by a marked decrease in testosterone production in response to hCG. This latter parameter may be viewed as a specific response of theca cells, which are a principal source of ovarian androgen since granulosa cells have minimal, if any, ability to produce androgens (29). Adrenal glands may also respond to hCG since LH/hCG receptors have been identified in human adrenal cortex in zona reticularis and fasciculata (30). However, in the present study, the key androgen secreted by adrenal glands, DHEAS, was not significantly altered by metformin treatment and, hence, testosterone response to hCG is most likely due to the effects on theca cells and not adrenal cells.

A particularly interesting and novel finding of the present study is the detection of a significant improvement in endotoxemia as evidenced by reduced levels of LPSs and LBP following metformin treatment. These effects may play a significant role in the improvement in ovarian hyperandrogenism. Indeed, LPSs have been shown to increase androgen production by stimulation of proliferation of theca cells and increasing expression of key enzymes involved in androgen synthesis (10). LBP is also highly relevant to LPS activity. It is an acute-phase reactant protein produced predominantly by the liver and capable of forming a complex with LPSs. This LPS–LBP complex activates Toll-like receptor 4 and ultimately stimulates inflammatory pathways. The importance of inflammatory pathways in regulation of androgen production has been demonstrated observations that an inhibition of these pathways by ibuprofen greatly reduces androgen production *in vitro* (in rat ovarian tissues) and *in vivo in* women with PCOS (31, 32). It is likely that the reduction in LPSs and LBP by metformin may be due, at least in part, to previously demonstrated effects on gut permeability (22, 23) or alterations of the gut microbiome (24, 25).

Another potential mechanism of action of metformin on the reduction in ovarian androgen production may be mediated by improvement in insulin resistance and consequent reduction in insulin (17, 33). Indeed, insulin can stimulate the growth of theca cells and increase the production of androgen (34, 35, 36).

Metformin may also affect ovarian androgen production by directly decreasing theca cell androgen synthesis (37) and by reducing the number of antral follicles (17, 38). The present study included a detailed evaluation of the number and size distribution of ovarian follicles; metformin treatment led to a selective decrease in the number of follicles measuring less than 6 mm in diameter. These observations are significant, since PCOS is specifically associated with an increase in the number of follicles measuring up to 6 mm (39).

In conclusion, the present study identified several mechanisms that may mediate the inhibitory effects of metformin on ovarian hyperandrogenism in women with PCOS. In particular, novel observations include a demonstration of reduction in endotoxemia and thus likely an anti-inflammatory action of metformin as well as an improvement in ovarian morphology characterized by a selective reduction in the number of smaller antral follicles. Future studies may focus on the determination of effects of metformin on gut microbiome and identification of mechanism mediating actions of metformin on folliculogenesis.

## Declaration of interest

The authors declare that there is no conflict of interest that could be perceived as prejudicing the impartiality of the work reported.

## Funding

This work did not receive any specific grant from any funding agency in the public, commercial or not-for-profit sector.
